# Exoproteome profiling of *Trypanosoma cruzi* during amastigogenesis early stages

**DOI:** 10.1371/journal.pone.0225386

**Published:** 2019-11-22

**Authors:** Samuel C. Mandacaru, Rayner M. L. Queiroz, Marcos R. Alborghetti, Lucas S. de Oliveira, Consuelo M. R. de Lima, Izabela M. D. Bastos, Jaime M. Santana, Peter Roepstorff, Carlos André O. Ricart, Sébastien Charneau

**Affiliations:** 1 Laboratory of Protein Chemistry and Biochemistry, Department of Cell Biology, Institute of Biology, University of Brasilia, Brasilia, Brazil; 2 Department of Biochemistry and Molecular Biology, University of Southern Denmark, Odense, Denmark; 3 Pathogen-Host Interface Laboratory, Department of Cell Biology, Institute of Biology, University of Brasilia, Brasilia, Brazil; Karolinska Institutet, SWEDEN

## Abstract

Chagas disease is caused by the protozoan *Trypanosoma cruzi*, affecting around 8 million people worldwide. After host cell invasion, the infective trypomastigote form remains 2–4 hours inside acidic phagolysosomes to differentiate into replicative amastigote form. *In vitro* acidic-pH-induced axenic amastigogenesis was used here to study this step of the parasite life cycle. After three hours of trypomastigote incubation in amastigogenesis promoting acidic medium (pH 5.0) or control physiological pH (7.4) medium samples were subjected to three rounds of centrifugation followed by ultrafiltration of the supernatants. The resulting exoproteome samples were trypsin digested and analysed by nano flow liquid chromatography coupled to tandem mass spectrometry. Computational protein identification searches yielded 271 and 483 protein groups in the exoproteome at pH 7.4 and pH 5.0, respectively, with 180 common proteins between both conditions. The total amount and diversity of proteins released by parasites almost doubled upon acidic incubation compared to control. Overall, 76.5% of proteins were predicted to be secreted by classical or non-classical pathways and 35.1% of these proteins have predicted transmembrane domains. Classical secretory pathway analysis showed an increased number of mucins and mucin-associated surface proteins after acidic incubation. However, the number of released trans-sialidases and surface GP63 peptidases was higher at pH 7.4. Trans-sialidases and mucins are anchored to the membrane and exhibit an enzyme-substrate relationship. In general, mucins are glycoproteins with immunomodulatory functions in Chagas disease, present mainly in the epimastigote and trypomastigote surfaces and could be enzymatically cleaved and released in the phagolysosome during amastigogenesis. Moreover, evidence for flagella discard during amastigogenesis are addressed. This study provides the first comparative analysis of the exoproteome during amastigogenesis, and the presented data evidence the dynamism of its profile in response to acidic pH-induced differentiation.

## Introduction

*Trypanosoma cruzi* is the etiologic agent of Chagas disease in Central and South America, with more than 10,000 deaths annually worldwide [1]. An increasing number of cases are being reported in non-endemic regions including the United States and Europe due to intense migration of individuals from endemic areas of Latin America [2–6]. In humans, *T*. *cruzi* infection usually develops from an acute phase characterized by high parasitemia and a robust immune response, into a clinically variable chronic phase. In the absence of treatment during the chronic phase, parasite proliferation is highly contained by a humoral and cellular immune response. However the infection remains persistent, particularly in the myocardium and smooth muscle, which may lead to the development of cardiac and digestive complications [7,8]. The treatment of Chagas disease is currently based around chemotherapy, since no effective vaccine is available. Only two drugs are available: nifurtimox and benznidazole. Benznidazole is considered the first-line treatment in most countries due to its effectiveness for the treatment of acute, congenital, reactive and early chronic infections. In many cases, this drug is able to reduce disease progression, but the high toxicity has the potential to cause serious side effects, leading to interruption of patient treatment. Additionally, the low efficacy of the current drugs is low in adult patients with the chronic disease [9,10].

In order to carry out its lifecycle, infective *T*. *cruzi* trypomastigotes invade nucleated mammalian host cells and differentiate intracellularly into replicative amastigote forms (amastigogenesis). After internalization, trypomastigotes remain 2–4 h inside acidic phagolysosomes [11], escaping into the host cell cytoplasm to complete its differentiation. To facilitate the escape process, the parasite recruits trans-sialidase proteins and release pore-forming molecules called Tc-TOX [12,13]. It has also been recently reported that *T*. *cruzi* infection blocks the expression of host cell immunoproteasome subunits, proteasome activator protein PA28b, TAP1 and MHC class I molecule by an unknown posttranscriptional control [14]. This indicates that trypomastigotes release proteins and/or other molecules in response to processes caused by or following the differentiation induction.

The secreted/excreted protein repertoire (here generically referred as exoproteome) plays important roles in homeostasis, immune response, development, proteolysis, adhesion, cell proliferation, cell differentiation, morphogenesis and cellular communication [15]. Furthermore, secreted/excreted proteins account for approximately 10% of the proteins encoded by a genome [15–18]. Trypomastigotes are able to release membranous vesicles filled with virulent factors such as trans-sialidases [19,20]. It is these extracellular vesicles that have been shown to be involved in the pathogenesis of Chagas disease by increasing heart parasitism and inflammation [20].

Classically secreted proteins can be identified by the presence of an N-terminal cleavable signal peptide (SP) that is typically 15–30 amino acids long. Furthermore, a class of secretory proteins, known as leaderless proteins, is exported from the cell without signal sequences through non-classical secretion pathways. For example cell surface shedding and inclusion into exosomes and other secretory vesicles [21], or even release from the plasma membrane through the enzymatic cleavage of their lipid anchor [22].

The acidic milieu is a key step in triggering amastigogenesis and parasite molecular response during this process and has been studied through high-throughput quantitative proteomic and phosphoproteomic approaches [23]. Queiroz and colleagues, analysing intracellular proteins 2 hours after induction, reported the overexpression of several proteins predicted to be secreted, indicating an increase in vesicular traffic. This observation leads us to hypothesize a change in parasite exoproteome repertoire after acidic-pH induction. To address this hypothesis, we evaluate the exoproteome changes of *T*. *cruzi* trypomastigote upon the first three hours of acidic-pH-induced axenic amastigogenesis compared to the exoproteome of trypomastigote incubated at physiological pH for the same period.

## Materials and methods

### Trypomastigote cell culture

Trypomastigotes, Y strain [24], were maintained in monolayers of HeLa cells grown in DMEM supplemented with 5% fetal bovine serum, at pH 7.4, according to [25,26]. The parasites of the outbreak from the 4th to 5th day after infection were carefully collected from the supernatant, consisting of over 98% trypomastigotes [26].

### Exoproteome samples

Trypomastigote cells were washed 3 times with DMEM, pH 7.4, without serum, by centrifugation at 2,500 × *g* for 10 min. Then, 1.0 × 10^9^ washed parasites were resuspended in 5 mL DMEM without serum at pH 7.4 or pH 5.0, (2.0 × 10^8^ cells/mL final concentration) and incubated in a 25 cm^2^ culture flasks at 37°C for 3 h with gently shaking every 20 min. After incubation, the parasites motility was microscopically monitored and the samples were collected only if ~ 95% of the cells remained mobile [27]. For pH 7.4 we obtained samples in duplicate and for pH 5.0 in triplicate. In order to remove cells following incubation, the medium was centrifuged for 5 min at room temperature in 3 rounds to ensure complete removal of cells and avoid mechanical cell lysis: firstly, at 2,000 × *g*, then at 4,000 × *g* and the last at 6,000 × *g*, with the supernatants transferred to new tubes after each centrifugation. After cell removal, the supernatants containing the exoproteomes were concentrated and buffer exchanged to 20 mM triethylammonium bicarbonate using Amicon^TM^ filter units with 3 kDa cut-off membrane (Millipore, Billerica, MA, USA), dried and stored at -20°C.

### Sample preparation for LC-MS/MS

The exoproteome samples were resuspended in 20 mM triethylammonium bicarbonate, reduced with 20 mM dithiothreitol at 56°C for 45 min, alkylated with 40 mM iodoacetamide in the dark at room temperature for 60 min and digested overnight at 37°C with 1 μg modified trypsin (Promega, Madison, USA). After digestion, the sample was acidified to 0.1% trifluoracetic acid (TFA), final concentration, and desalted with homemade microcolumns of Poros Oligo R3 resin (PerSeptive Biosystems, Framingham, USA) packed (1 cm long) in p200 tips (adapted from [28]). Prior to lyophilization, a Biochrom 30 amino acid analyzer (Biochrom, Cambridge, U.K.) was employed to determine peptide concentration according to the manufacturers protocol [29].

### LC–MS/MS and data analysis

Samples were analysed by an EASY-nano LC system (Proxeon Biosystems, Odense, Denmark) coupled online to an LTQ-Orbitrap Velos mass spectrometer (Thermo Scientific, Waltham, USA). The exoproteomes at physiological pH and acidic pH were analysed in duplicate and in triplicate, respectively. Two μg of peptides from each fraction were loaded onto an 18 cm fused silica emitter (75 μm inner diameter) manually packed with reverse phase capillary column ReproSil-Pur C18-AQ 3 μm resin (Dr. Maisch GmbH, Germany) and eluted using a gradient from 100% phase A (0.1% formic acid) to 35% phase B (0.1% formic acid, 95% acetonitrile) for 210 min for each sample, 35% to 100% phase B for 5 min and 100% phase B for 8 min in (a total of 223 min at 250 nL/min) [29]. After each run, the column was washed with 90% phase B and re-equilibrated with phase A. Mass spectra were acquired in positive ion mode applying data-dependent automatic survey MS scan and tandem mass spectra (MS/MS) acquisition. Each MS scan in the orbitrap (mass range of m/z of 400–1800 and resolution 60,000) was followed by MS/MS of the seven most intense ions in the LTQ. Fragmentation in the LTQ was performed by HCD and selected sequenced ions were dynamically excluded for 30 s. Raw data were viewed in Xcalibur v.2.1 (Thermo Scientific, Waltham, USA). Data processing was performed using Proteome Discoverer v.1.3 (Thermo Scientific, Waltham, USA). Raw files were generated, and these were searched using Proteome Discoverer with SequestHT algorithm against *Trypanosoma cruzi* database containing the proteins of the parasite reference proteome database downloaded from UniProt (early 2017). Contaminant proteins (several types of human keratins, BSA and porcine trypsin) were also added to the database and all contaminant proteins identified were manually removed from the result lists. The searches were performed with the following parameters: MS accuracy 10 ppm, MS/MS accuracy 0.5 Da, trypsin digestion with up to 2 missed cleavage allowed, fixed carbamidomethyl modification of cysteine and variable modification of oxidized methionine. The number of proteins, protein groups and number of peptides were filtered for a false discovery rate (FDR) less than 1%; peptides with rank 1 and proteins with at least 3 peptides using Proteome Discoverer. ProteinCenter^™^ software (Thermo Scientific, Waltham, USA) was used to generate FASTA formatted files of groups of proteins of interest, GO annotation and statistical analysis between conditions (Fisher's exact test). Improved annotation of the identified proteins was acquired using Blast2GO software (http://www.blast2go.com/b2ghome) using default parameters. SignalP v.4.1 (http://www.cbs.dtu.dk/services/SignalP/) and SecretomeP v.2.0 (http://www.cbs.dtu.dk/services/SecretomeP/) were used to predict proteins secreted by classical and non-classical pathways, respectively. The parameters, eukaryotes/mammal, gram positive and gram negative were set to predict the secretion pathways. The TMHMM algorithm (http://www.cbs.dtu.dk/services/TMHMM/) was used to predict the number of transmembrane helixes in the protein sequences.

## Results and discussion

**Fig 1:**
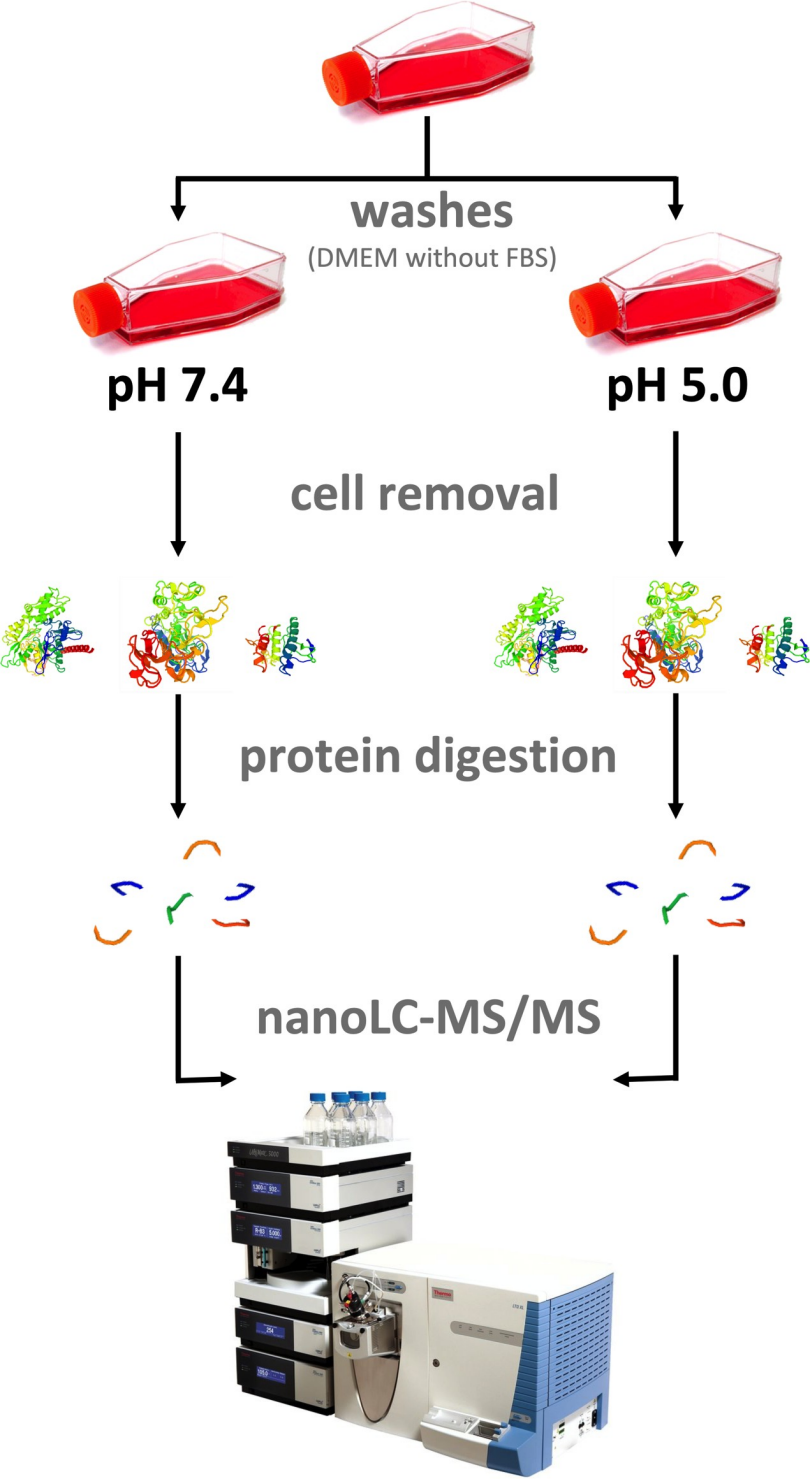
Experimental setup. Tissue culture-derived trypomastigotes were harvested and washed before incubation in DMEM without FBS at pH 7.4 or pH 5.0 for 3 h. After incubation, the parasites were removed by three cycles of centrifugation and the proteins presented in the supernatant were TCA/acetone precipitated. Following protein digestion, peptides were subjected to nanoLC-MS/MS analysis.

**Fig 2:**
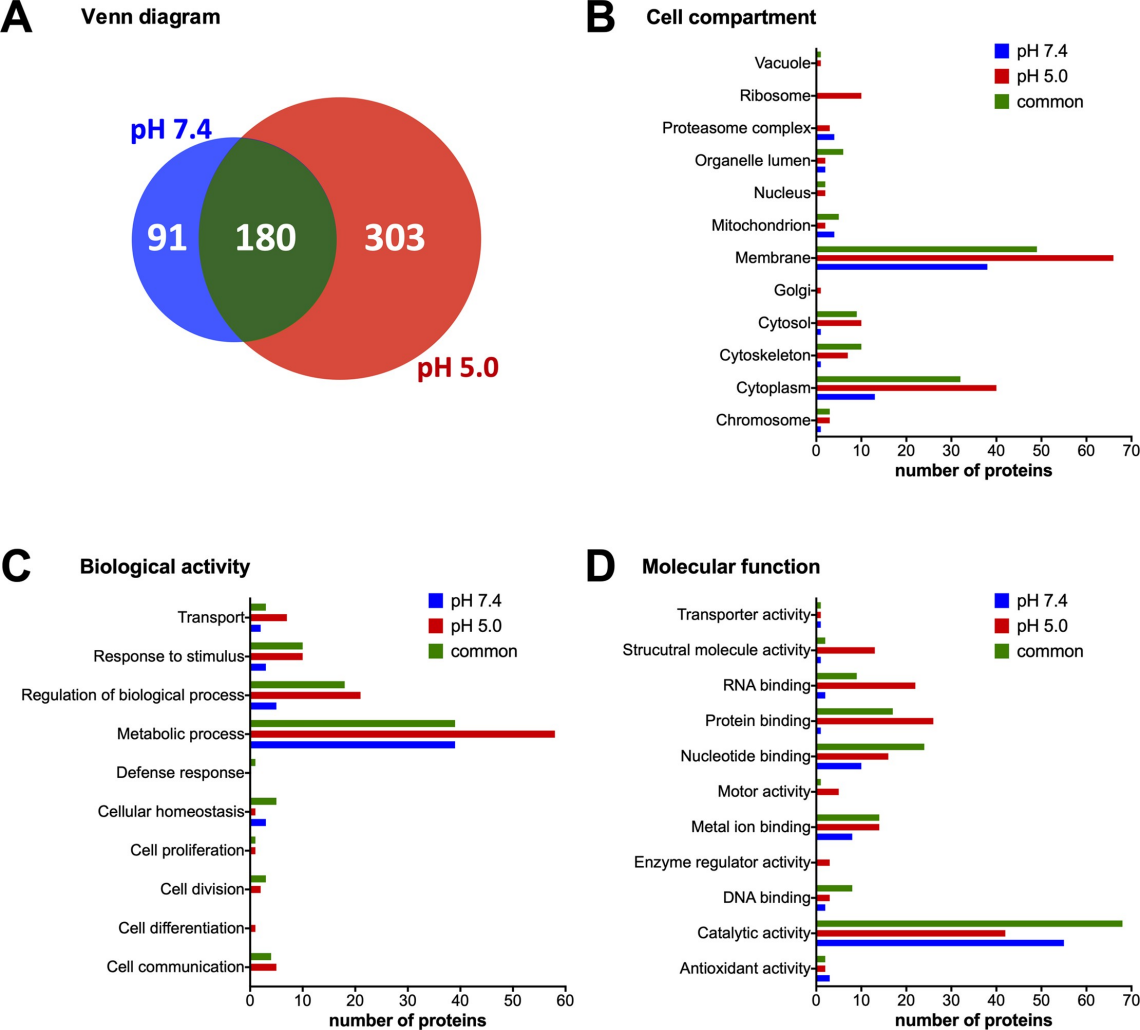
Comparison of *T*. *cruzi* trypomastigote exoproteome at pH 7.4 and pH 5.0. Venn diagram of protein groups identified in each condition (**A**). Protein categorization by GO annotation for cell compartment (**B**), molecular function (**C**) and biological activity (**D**). Y-axis represents the number of proteins present in each GO term.

In order to identify proteins secreted by *T*. *cruzi* during amastigogenesis, we performed a qualitative exoproteome analysis of trypomastigote in two different conditions. Thus, samples from parasites incubated for 3 hours at pH 7.4 (control) or at pH 5.0 (amastigogenesis) were investigated by shotgun/bottom-up proteomics (Fig 1). The computational analysis of LC-MS/MS data identified 271 and 483 protein groups at pH 7.4 and pH 5.0 respectively, with 180 common protein groups being present in both conditions (Fig 2A; S1 Table).

The acidic pH-induced differentiation causes drastic metabolic and morphologic changes in *T*. *cruzi* trypomastigotes [23], preparing the parasite for a replicative stage. As demonstrated by Engel et al. (1985), the amastigote pre-replicative lag period spans from 18.2 to 34.2 hours, depending on the cloned stock analyzed. In 1995, Tomlinson *et al*. showed that, 2 hours after pH induction, almost no trypomastigotes were observed in culture and 4h after pH induction, more than 90% of trypomastigotes were transformed into amastigotes. Focusing on the transformation phase of this parasite, the exoproteome was analyzed at a time point of 3 hours after pH-induced differentiation. After this time, the exoproteome reflects changes in the trypomastigote transformation to amastigote and not the amastigote exoproteome "*per se*". In fact, amastigogenesis dynamics after pH lowering was also evaluated by Hernández-Osorio [30] and they found that, after 3 hours, intermediate forms were predominant (over 80%). Therefore, the exoproteome analysed here can be related to early trypomastigote morphological changes, microenvironment modulation to the next replicative phase and host cell metabolism modulation to parasite survival and replication. In 2013, Caradonna et al. [31], demonstrated that several host metabolism and cytoskeleton modulations which support parasite intracellular growth. Energy production, nucleotide metabolism, pteridine metabolism and fat acid oxidation were shown as interconnected pathways between host and parasite, regulated by host Akt signaling. The capability of adaptation would be especially relevant in the context of a natural dynamic infection in the mammalian host as observed during amastigogenesis. Plasticity within the same population could reflect the ability to change the environment in order to control growth rates. Some specific microenvironments can be a critical issue underlying tissue tropism and persistent infection [31]. An example of this can be observed during *T*. *cruzi* infection in adipose tissue and muscle. The fatty acid rich environment and high metabolism of energy production of these cells, provide a space against immune system [31]. The total amount and diversity of proteins released by the parasites almost doubled upon acidic induction within the three hours of incubation (Fig 2A). While hundreds of proteins were identified in both conditions, a significant number of identified proteins were specific for each one (Fig 2A). While 33.6% (91/271) of proteins were specific of pH 7.4 exoproteome, 62.7% (303/483) were specific of pH 5.0 condition (S1 Table). It suggests that there is an increase in protein diversity in exoproteome during amastigogenesis.

Overall, at pH 5.0 all GO categories increased except for proteasome complex and mitochondrion (cell compartment), cellular homeostasis (biological activity) and catalytic activity and antioxidant activity (molecular function) (Fig 2B–2D). Fisher's exact test between conditions also showed proteins with catalytic activity and hydrolase activity (a subcategory of catalytic activity) under-represented at pH 5.0 (Table 1). Fig 2B highlights the common membrane components in exoproteome, particularly at pH 5.0—indicating potential parasite surface remodeling. Metabolic process, regulation of biological process, response to stimulus and transport (Fig 2C) are the most represented term of the biological activity category. In terms of molecular function, the high number of proteins with catalytic activity corroborate the myriad of metabolic processes in the exoproteome (Fig 2D). Altogether, it is likely that the parasite renews its metabolism during the first hours of amastigogenesis.

**Table 1:** Under-represented GO terms in parasites incubated at pH 5.0 compared to pH 7.4.

	Description	Count ^*a*^	Ref. Count ^*b*^	Raw p-value ^*c*^	FDR p-value ^*d*^
**Molecular Function**	catalytic activity	113	125	4.10E-07	2.75E-04
	hydrolase activity	63	78	6.23E-05	2.09E-02

^*a*^ number of times this feature occurs in the analysis data set.

^*b*^ number of times this feature occurs in the reference data set.

^*c*^ raw p-value indicating the significance of this difference in feature occurrence between the data sets.

^*d*^ FDR corrected version of the raw p-value.

**Fig 3:**
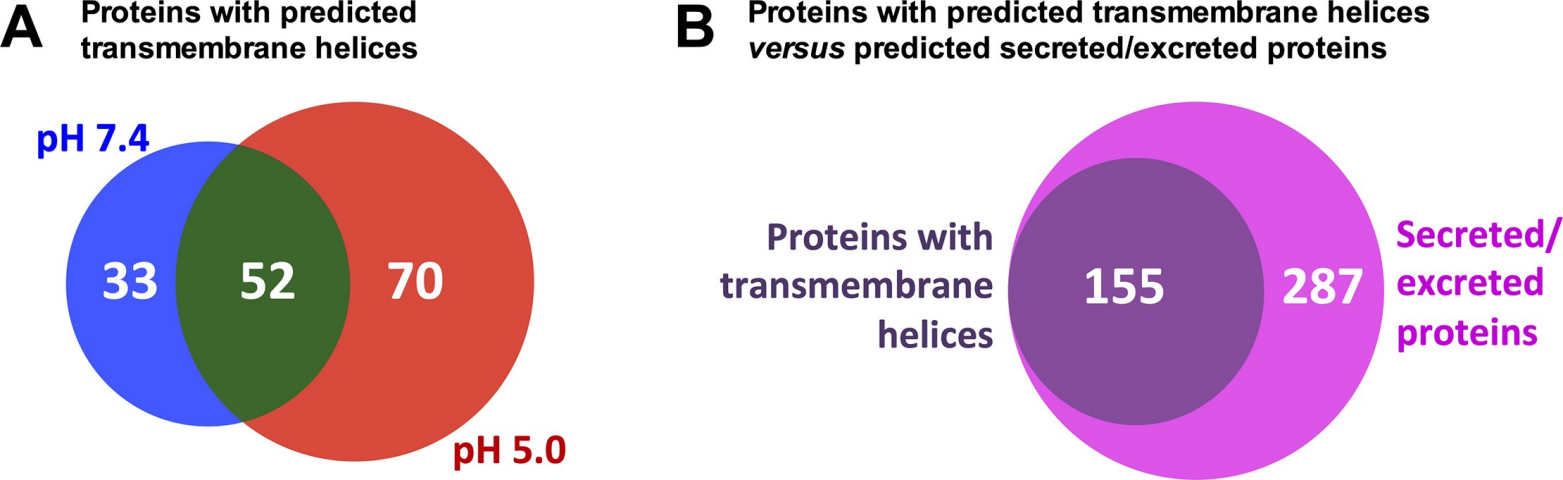
TMHMM analysis. Increased number of proteins predicted to possess transmembrane domain in pH 5.0 related to pH 7.4 (A) and all proteins with predicted transmembrane domains are also predicted to be secreted/excreted (B).

Secretome analysis of *T*. *cruzi* epimastigotes and metacyclic trypomastigotes reported a considerable amount of microvesicles and exosomes [32]. The parasite has different strategies to mediate intercellular communication [33,34], and these vesicles can be used to interact directly with host cells by transferring several small molecules such as proteins, mRNAs, microRNAs and small molecules [33]. Vesicles can transport proteins in soluble form, associated or as integral components of membranes. Transmembrane domains were predicted in 27% of proteins (155/574), with 33 proteins exclusively detected at pH 7.4, 70 at pH 5.0 and 52 in both conditions. These proteins presented up to four predicted transmembrane helices (Fig 3A). Based on this analysis, our results indicate that cell-derived trypomastigotes in both pH conditions could also release vesicles. Moreover, the increased proportion of transmembrane proteins in the exoproteome at pH 5.0, compared to pH 7.4 condition, indicates that transition of trypomastigotes to the early stage of amastigotes may trigger other types of secretion/excretion besides vesicles. However, further experiments to confirm this hypothesis are necessary. All 155 proteins with predicted transmembrane domains were also predicted to be secreted (Fig 3B), suggesting that these proteins could be present in vesicles or being secreted/excreted.

**Fig 4:**
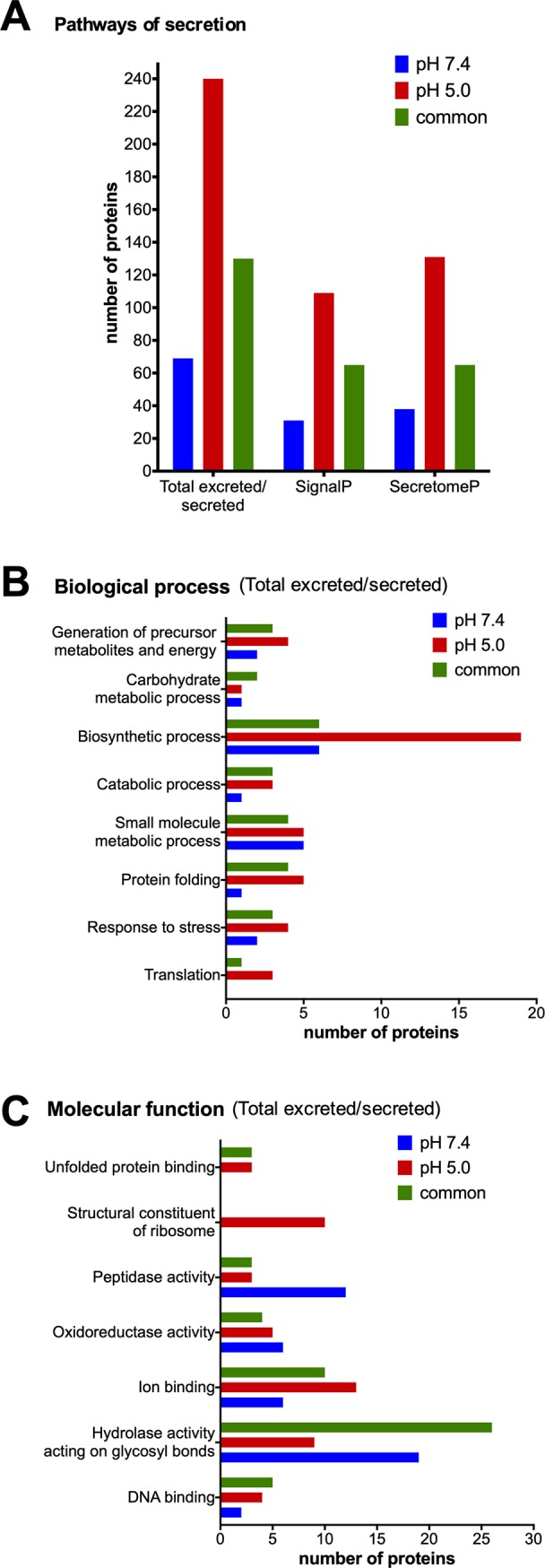
Secretory pathway prediction. Total number of predicted proteins secreted and by classical (SignalP) and non-classical pathways (SecretomeP) **(A)**. Blast2GO annotation of *T*. *cruzi* trypomastigote exoproteome at pH 7.4 and pH 5.0 and comparison of most abundant biological activity (**B**) and molecular function GO terms (**C**) of annotated secrete/excreted proteins.

*In silico* screening of excreted/secreted proteins based on genomic information cannot be considered self-sustaining evidence for its secretion as the prediction accuracy is highly dependent on the tool performance and quality of the genomic annotations. Furthermore, predicted secretory proteins may not be expressed in the particular cell/condition under examination or have a retention signal that prevents their secretion [15]. Integrating bioinformatic analysis to predict secreted proteins with proteomic data reinforce the excreted/secreted status of a particular protein and provides validation for the subproteome enrichment and its quality. Herein, *in silico* prediction of secretion through classical (SignalP) and non-classical pathways (SecretomeP) of trypomastigote exoproteomes at pH 7.4 and pH 5.0 showed remarkably that 76.5% (439/574) of all detected proteins are predicted to be secreted in both algorithms (Fig 4A). Almost twice the number of proteins predicted to be secreted was identified in the acidic condition (199 proteins at pH 7.4 *versus* 370 proteins at pH 5.0, Fig 4A). This corroborates the hypothesis raised from previously published findings on over expression of several proteins (e.g. some Rab proteins and ADP-ribosylation factors), within 2 hours of acidic induction, indicating an increase in vesicular traffic [23]. In addition, most proteins were predicted to be released through non-classical pathways at pH 5.0 (131 versus 109). While a modest difference, it is in accordance with other findings for members of the kinetoplastidae order [35–37]. Despite computational prediction presents some issues (e.g. none of the algorithms were designed specifically for Trypanosomatids), a very high percentage of predicted proteins together with the GO annotation profiles are concordant synergic and ratify our sampling and results.

Kinetoplastids database was explored with *Blast2GO* software in order to characterize the total of proteins predicted to be secreted/excreted. The biological processes: translation, response to stress, protein folding, biosynthetic process, generation of precursor metabolites and energy displayed higher number of proteins at pH 5.0 (Fig 4B). DNA binding, ion binding, structural constituent of ribosome and unfolded protein binding were molecular functions predominantly represented at pH 5.0 and hydrolase activity acting on glycosyl bonds and peptidase activity were under represented in this condition (Fig 4C). As expected, the most assigned term was *hydrolase activity on glycosyl bonds* due to the extent of trans-sialidases family members.

**Fig 5:**
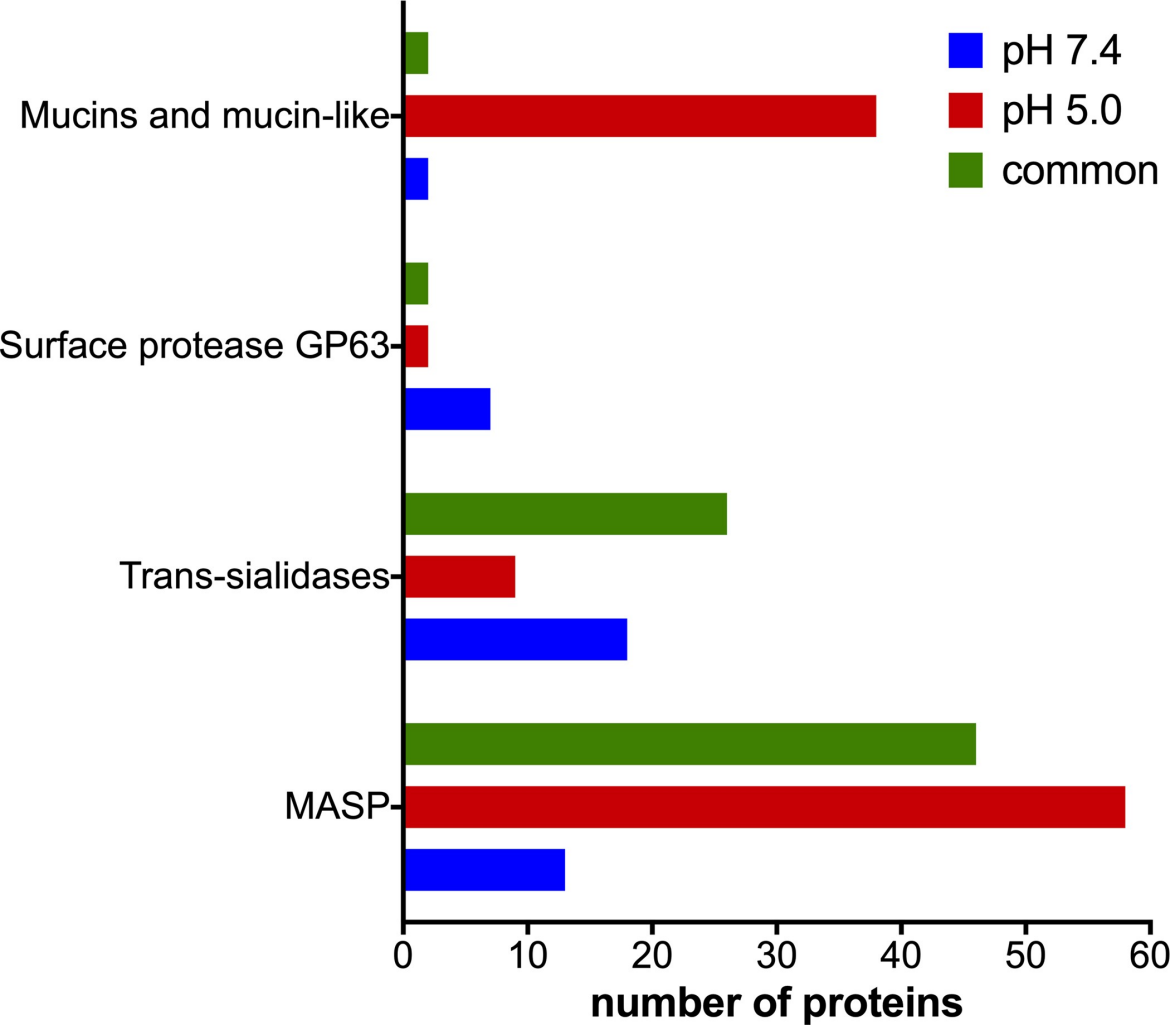
Representative proteins from classical secretory pathway in *T*. *cruzi*. Most abundant proteins released through classical pathway within *T*. *cruzi* trypomastigote exoproteome at pH 7.4 and pH 5.0.

The most abundant proteins identified on classical secretory pathway analysis are shown on Fig 5. Trans-sialidases (TS) are enzymes that transfer sialic acid to mucins and both macromolecules are anchored to the plasma membrane by glycosylphoshatidylinositol [38,39]. This enzymatic reaction promotes protection to the parasite against the host immune system and promotes cell invasion. TS function is also important for parasitic escape from parasitophorous vacuole. The number of trans-sialidases identified in acidic conditions was lower than at pH 7.4 (35 and 44, respectively) (Fig 5). Furthermore, specific trans-sialidases were identified in each condition, as previously reported [40]. Specific trans-sialidases, at pH 5.0, could be involved in parasitophorous escape and, at pH 7.4, could be involved in immune system escape and cell invasion (Fig 5).

Mucins repertory changes along parasite cell cycle [22] and, as expected, striking identification differences between both conditions for mucin and mucin-like proteins were observed. These families jumped from 2 representatives in the exoproteome at pH 7.4 to 38 different proteins at pH 5.0. Interestingly, a transcriptomic analysis showed a decrease in mRNA for mucins during amastigogenesis and an increase of transcripts for membrane-bound/secreted phospholipase A1 [41] and for surface-localized phosphatidylinositol-phospholipase C (PI-PLC) [42]. This increase in phospholipase transcripts may explain the increase of mucins in this secretome analysis [43]. Similarly, a study performed an analysis of glycoconjugate mucin secretion in cultured rat conjunctival goblet cells, and observed an increase of mucin secretion directly related to the phospholipase C and phospholipase A2 dependent-Ca^2+^ performance under physiological conditions [43]. This mechanism may be employed as the same manner in *T*. *cruzi* parasites, considering that many cellular processes are conserved among eukaryotes. This data provides evidence that the parasite remodels the cell coat releasing surface proteins during amastigogenesis.

Mucin-associated surface proteins (MASP) exhibited the same pattern of mucins with a considerable increase in acidic pH, from 13 proteins identified specifically at pH 7.4 to 58 specifically at pH 5.0 and 56 in both conditions. The *T*. *cruzi* genome has approximately 1,300 clustered genes coding for MASPs and these proteins can be shed by circulating and infective parasites [44,45]. Using anti-MASP antibodies, Bartholomeu et al., (2009), detected MASP in the supernatant of PI-PLC treated trypomastigotes [44]. In this scenario, increased MASP levels after acidification condition could be explained by increasing action of phospholipases, in the same fashion for mucins, even with downregulation of MASP transcripts during early amastigote development [46]. In addition, few members of these families (trans-sialidases, mucins and MASP) were also predicted to be secreted through non-classical pathway.

In addition, some heat shock proteins (HSPs) and HSP-associated proteins, such as chaperonins, co-chaperones, prefoldins, calreticulin and cyclophilins or peptidyl prolyl isomerases were found in both exoproteome conditions. In the acidic environment, 13 HSPs and HSP-associated proteins were detected, and some of them playing important roles in the macrophage activity. For example, Hsp10 inhibits classical LPS-induced activation of macrophages due to pro-inflammatory cytokine synthesis [47,48]. *T*. *cruzi* parasites also infect macrophages, and require an ideal environment to replicate, nonetheless, this mechanism needs to be experimentally demonstrated in this organism. Moreover (change), Hsp10 is considered a circulating anti-inflammatory factor that possibly acts to contain macrophage activation [49].

Conversely, in both conditions, cyclophilins or peptidyl prolyl isomerases, Hsp70 and clusterin are involved in the classically activation of macrophages [50]. In *Toxoplasma gondii*, cyclophilin-18 induced IL-12 production by dendritic cells and triggered cell signalling through CCR5. This mechanism may provide a strong protective response to the parasite allowing its transmission, avoiding host’s intermediates [51]. Interestingly, Hsp70 of *Mycobacterium tuberculosis* inhibits the infection of CD4+ T cells by HIV-1, blocking the CCR5 co-receptor [52] in other words, despite its role as a chaperone in the activation of macrophages [50] and protein folding, this protein may help the parasite in its survival and proliferation, inhibiting invasion of competitive microorganisms. Further, clusterin is known as a secreted extracellular chaperone capable to bind unfolded proteins, which could promote receptor-mediated endocytosis and intracellular lysosomal degradation [53]. Likewise, prefoldin, only found at pH 5.0, seems to act as a co-chaperone mediating chaperone-substrate interactions [54]. These observations seem to be feasible to occur *in vivo*, considering the high acidic environment of phagolysosome and the whole arsenal of *T*. *cruzi* HSP and HSP-associated proteins.

Since proteases are involved in crucial steps of the biological life cycle of *T*. *cruzi* including aspects of host-parasite interaction, these enzymes are subjects of special attention [55–57]. The most studied zinc-dependent metalloproteases, also termed as GP63 family in trypanosomatids, are described as major surface glycoproteins with acid protease activity and virulence factors [58,59]. GP63 genes are present at high-copy-number [60] and encode proteins involved in parasite-host interaction. After 3 hours of incubation, the number of specific identifications at pH 5.0 and pH 7.4 was 2 and 7, respectively and 2 were present in both conditions. GP63, MASP, mucin-like proteins are surface membrane proteins that compose extracellular vesicles of trypomastigote [61] and of the early stages of amastigote differentiation accordingly to our data. Besides its ability to interact with the host extracellular matrix, GP63 is also able to inhibit NK cellular function. In trypanosomatids, this promotes resistance to antimicrobial peptides, intracellular amastigote survival in macrophages and degradation of cytosolic proteins of host cells. Altogether it demonstrates the versatility of GP63 in parasite survival in conditions of stress [62–64].

Calpain-like proteins is another family of proteins with a large number of different genes present in trypanosomatid genomes [65]. Calpains are found as microtubule-interacting proteins in *T*. *cruzi and T*. *brucei* [56]. Two calpain-like cysteine peptidases were only released at pH 7.4 as well as at pH 5.0, respectively, and 2 more in both conditions. One calpain peptidase and the calpain-like CAP5.5 (cytoskeleton-associated protein 5.5) were recently described as immunoreactive proteins recognized by serum immunoglobulin from chagasic patients with early cardiomyopathy [66]. Moreover, CAP5.5 was shown to be secreted/excreted by metacyclic trypomastigotes [32]. According to our data, calpain cysteine peptidases and CAP5.5 are released (associated or not to vesicles) during the trypomastigote differentiation into amastigote.

Several proteins related to ubiquitin signaling were identified from the exoproteome at pH 5.0 and pH 7.4 (S2 Table). None of them were classified as secreted through classical pathway. For the parasites, ubiquination and ubiquitin-proteasome pathway are crucial in key steps in host colonization (proliferation and cell differentiation) [67] and for the host cells to modify immunoregulatory functions [68]. Bacteria and viruses secrete ubiquitin signaling related proteins into host cell. In *T*. *cruzi*, a protein related to ubiquitin signaling was shown to be secreted into the host cell and to localize in the nucleus [69]. Our data suggests that such proteins may function in the host cell nuclei orchestrating host regulatory elements towards parasite survival inside the host cell.

Intraflagellar vesicle transport occurs via microtubules driven by motors such as proteins belonging to the kinesin and the dynein family [70]. SNF-7 is a protein related to cargo transport through cytoskeleton and vesicle coating, which was secreted at pH 5.0 conditions only. Secretion profiles of transport and vesicle structures may indicate a dynamic parasite behaviour including an actively remodelling intracellular expression pattern related to the exportation of molecules during early stages of amastigogenesis. It fits with the idea that after cell invasion, trypomastigote disassemble and discard their flagella into host cell cytoplasm where it is degraded. This process releases flagellar proteins that enter the MHC-I processing pathway and presentation to CD8+ T cells as demonstrated for paraflagellar rod protein PAR4. Additionally, it was demonstrated that TcPAR4 immunization in mice enhanced resistance to *T*. *cruzi* [71]. Our results corroborate previous observations that TcPAR4 is released after parasite cell invasion. Paraflagellar rod component Par4 putative (Q4CUM0) was found exclusively in the parasite exoproteome at pH 5.0 (Table 2). This work provides 22 flagellar proteins exclusively identified at pH 5.0 and 12 proteins shared at pH 5.0 and pH 7.4. Among these proteins, kinesins and dyneins (or associated proteins) were found only at pH 5.0 or shared in both conditions. Strikingly, no flagellar proteins were identified as being released exclusively at pH 7.4. As demonstrated to TcPAR4, these proteins can be candidates for vaccines or good protein targets for new chemotherapy strategies.

**Table 2:** Flagellar and flagellar-associated proteins in the *T*. *cruzi* exoproteome.

UniProt	Description	pH 7.4	pH 5.0
	*Secreted proteins only detected from amastogogenesis early stages*		
Q4D2I4	putative ADP ribosylation factor 3		X
Q4DS99	paraflagellar rod component, putative		X
Q4D0Q5	flagella associated protein		X
Q4CQP1	putative STOP axonemal protein		X
Q4DG71	putative Flagellar attachment zone protein 1		X
Q4D1B7	putative paraflagellar rod component		X
Q4CUM0	paraflagellar rod component Par4, putative		X
Q4DRP5	flagellar pocket cytoskeletal protein bilbo1		X
Q4DWL5	paraflagellar rod component		X
Q4D113	flagellar member 7		X
Q4D8M9	putative paraflagellar rod proteome component 9		X
Q4DHQ3	flagellar radial spoke protein-like, putative		X
Q4DSB9	T. brucei spp.-specific protein		X
Q4CR32	hypothetical protein		X
Q4CUF2	flagellar protofilament ribbon protein, putative		X
Q4DRF1	putative paraflagellar rod component		X
Q4DZQ3	putative flagellar antigen		X
Q4DG38	putative dynein-associated protein		X
Q4DFG6	kinesin-like protein		X
Q4E1M8	kinesin, putative		X
Q4DYM0	kinesin, putative		X
Q4DWH2	dynein, putative		X
Q4DCS6	outer dynein arm docking complex protein		X
	*Secreted proteins detected from trypomastigote and amastogogenesis early stages*		
Q4DQ49	centrin, putative	X	X
Q4CTX0	flagellar calcium-binding 24 kDa protein	X	X
Q4DQS9	Flagellar attachment zone protein 10	X	X
Q4D7Y4	kinetoplastid membrane protein 11	X	X
Q4D634	paraflagellar rod protein 2	X	X
Q4DGZ9	flagellar member 3	X	X
Q4DUG1	flagellar member 3	X	X
Q4DIP8	flagellar associated protein	X	X
Q4CP97	putative mitochondrial paraflagellar rod component (PFC16)	X	X
Q4DIF6	paraflagellar rod protein 2	X	X
Q4D4E6	dynein intermediate chain, putative	X	X
Q4E2Q5	putative OSM3-like kinesin	X	X

## Conclusion

Acidic-pH-induced axenic amastigogenesis creates a lysosome-like environment, mimicking conditions when the parasite enters into the host cell. Exploiting this model, exoproteome analyses of early stages of amastigogenesis allowed the identification of several exclusive proteins at pH 5.0 related to cell communication, response to stimulus, regulation of biological process and cell division. In this scenario, exclusive proteins identified at pH 5.0 have the potential to modulate host cellular metabolism, allowing parasite survival, differentiation and proliferation. *T*. *cruzi* exoproteome changes during its life stage may provide advantages to parasites over host. Regarding trypomastigotes maintained in pH 7.4 or pH 5.0 for 3 hours, this is the first study to investigate the *T*. *cruzi* exoproteome change during the amastigogenesis. Our data provide evidence and direction for further studies to explore exoproteome changes during the first hours of amastigogenesis; highlighting the increase in number and diversity of proteins in acidic condition. This corroborates previous studies on the increase in vesicular trafficking during amastigogenesis. Furthermore, this work provides a list of vesicular and flagellar proteins released after acidic induction that could be explored as potential candidates to multitarget vaccines.

## Supporting information

S1 Table Proteins identified exclusively at pH7.4, at pH 5.0 and in both conditions. (XLSX)

S2 Table *Trypanosoma cruzi* proteins related to ubiquitin signaling and ubiquitin-proteasome pathway from exoproteome at pH 5.0 and pH 7.4. (PDF)
