# Supplementary material for: Exoproteome profiling of Trypanosoma cruzi during amastigogenesis early stages
Source: PLoS One. 2019 Nov 22;14(11):e0225386. doi: 10.1371/journal.pone.0225386 (PMC6874342; doi:10.1371/journal.pone.0225386)
Supplement: S2 Table — (PDF) [file pone.0225386.s002.pdf]

S2 Table. *Trypanosoma cruzi* proteins related to ubiquitin signaling and ubiquitin-proteasome pathway from exoproteome at pH 5.0 and pH 7.4.

**UniProt**	**Description**	**pH 5.0**	**pH 7.4**	**non-classical pathway secretion prediction**
Q4D439	ubiquitin-conjugating enzyme-like	X		YES
Q4DIR8	small ubiquitin protein	X		YES
Q4CPK3	ubiquitin-like protein	X		YES
Q4D4W4	putative mitochondrial ubiquitin fold modifier protein, putative (UFM1)	X		NO
Q4D144	proteasome alpha 3 subunit, putative	X		NO
Q4D1H4	proteasome alpha 5 subunit	X		NO
Q4CSX5	proteasome regulatory non-ATPase subunit	X		NO
Q4DSS5	small ubiquitin protein		X	YES
Q4E4G8	proteasome alpha 1 subunit		X	NO
Q4DIK4	proteasome alpha 3 subunit, putative		X	NO
Q4DW25	proteasome beta-1 subunit		X	NO
